# Post-resettlement context matters: a qualitative study of refugee parents’ responses to child trauma in low-resource settings

**DOI:** 10.1080/20008066.2026.2693362

**Published:** 2026-07-21

**Authors:** Mohsen Rajabi, Rachel Calam, Fatemeh Bagherifard, Victoria Williamson, Fatemeh Ashrafi, Anke Karl, Sarah L. Halligan

**Affiliations:** a Department of Psychology, University of Bath, Bath, UK; b Division of Psychology and Mental Health, University of Manchester, Manchester, UK; c Institute of Child Health, University College London, London, UK; d Institute of Psychology, Psychiatry and Neuroscience, King's College London, London, UK; e Association for the Protection of Refugee Women and Children (HAMI), Tehran, Iran; f Psychology, Faculty of Health and Life Sciences, University of Exeter, Exeter, UK; g Department of Psychiatry, Stellenbosch University, Stellenbosch, South Africa; h Department of Psychiatry and Mental Health, University of Cape Town, Cape Town, South Africa

**Keywords:** PTSD, refugee parents, post-resettlement context, child trauma, parenting, mental health, Iran, TEPT, padres refugiados, contexto posterior al reasentamiento, trauma infantil, crianza, salud mental, Irán

## Abstract

**Background:** Despite evidence that post-resettlement socio-environmental factors affect parenting, refugee parents’ experiences of meeting children’s posttrauma needs in resource-poor settings remain severely underexplored in LMICs.

**Objective:** This study aimed to explore parental understanding of child posttrauma distress and caregiving responses in the context of displacement and resource limitations in Iran, one of the world’s largest refugee-hosting countries.

**Methods:** Thirty refugee parents were recruited from deprived communities in Iran. Data were collected using semi-structured interviews and questionnaires. Parents were mainly from Afghanistan, most were illiterate, and all reported at least moderate PTSD symptoms. Data were analysed using thematic analysis.

**Results:** Four themes were generated regarding: (1) parents’ understanding and interpretation of children’s distress; (2) parenting under displacement; (3) intra-family dynamics; and (4) support needs, help-seeking, and care pathways. Parents lacked a basic understanding of their child’s trauma-related difficulties, describing extreme uncertainty about how to respond to their child’s emotional needs, as well as limited insight into those needs, coupled with a severely limited capacity to support their children and a lack of access to wider resources. Ongoing post-resettlement stressors appeared to deplete parents’ coping capacity and trigger harsh, reactive parenting, in contrast to more supportive parenting practices described before displacement. Material deprivation also influenced parent–child interactions: many children withheld trauma-related mental or physical consequences to avoid healthcare costs, and some adopted parenting-like roles, including income generation, sibling care, and emotional support for their distressed parents. Finally, parents’ priorities were wholly focused on material needs essential for their children’s basic survival, rather than psychosocial support.

**Conclusion:** Our findings provide novel insight into the profound influence that markedly low mental health literacy, material deprivation, and limited internal and external support resources have on how refugee parents understand and respond to their child’s posttrauma needs in deprived, low-resource post-resettlement settings.

## Highlights

- We captured perspectives from a historically under-represented refugee population in Iran.
- Lack of informational, material and internal resources significantly impacted parenting under post-resettlement adversity.
- Markedly low mental health literacy left parents uncertain about *when* and *how* to respond to their child’s posttrauma distress.
- Post-resettlement stressors were a key contributor to harsher and more reactive parenting.
- In resettlement communities, relatives and schoolteachers are key actors in helping refugee parents overcome acculturation stress.

## Abstract (es)

**Antecedentes:** A pesar de la evidencia de que los factores socioambientales posteriores al reasentamiento afectan la crianza, las experiencias de los padres refugiados para satisfacer las necesidades postraumáticas de sus hijos en entornos de escasos recursos siguen estando gravemente subexploradas en los países de ingresos bajos y medios (PIMB).

**Objetivo:** Este estudio tuvo como objetivo explorar la comprensión parental del malestar postraumático infantil y las respuestas de cuidado en el contexto del desplazamiento y las limitaciones de recursos en Irán, uno de los países que alberga a una de las mayores poblaciones de refugiados del mundo.

**Métodos:** Se reclutó a treinta padres refugiados de comunidades desfavorecidas en Irán. Los datos se recopilaron mediante cuestionarios y entrevistas semiestructuradas. Los padres procedían principalmente de Afganistán, la mayoría eran analfabetos y todos reportaron al menos síntomas moderados de TEPT (Trastorno de Estrés Postraumático). Los datos se analizaron mediante análisis temático.

**Resultados:** Se generaron cuatro temas relativos a: (1) la comprensión e interpretación de los padres sobre el malestar de sus hijos; (2) la crianza bajo la situación de desplazamiento; (3) la dinámica intrafamiliar; y (4) las necesidades de apoyo, la búsqueda de ayuda y las rutas de atención. Los padres carecían de una comprensión básica de las dificultades de sus hijos relacionadas con el trauma, describiendo una incertidumbre extrema sobre cómo responder a sus necesidades emocionales, así como una capacidad de introspección limitada respecto a dichas necesidades, sumado a una capacidad severamente limitada para apoyar a sus hijos y una falta de acceso a recursos más amplios. Los estresores continuos posteriores al reasentamiento parecieron agotar la capacidad de afrontamiento de los padres y desencadenar una crianza dura y reactiva, en contraste con las prácticas de crianza más comprensivas descritas antes del desplazamiento. La privación material también influyó en las interacciones entre padres e hijos: muchos niños se guardaron las consecuencias físicas o mentales relacionadas con el trauma para evitar costes sanitarios, y algunos asumieron roles parentalizados, incluyendo la generación de ingresos, el cuidado de hermanos y el apoyo emocional a sus padres afectados. Finalmente, las prioridades de los padres se centraron por completo en las necesidades materiales esenciales para la supervivencia básica de sus hijos, en lugar del apoyo psicosocial.

**Conclusión:** Nuestros hallazgos aportan una perspectiva novedosa sobre la profunda influencia que una alfabetización en salud mental marcadamente baja, la privación material y los limitados recursos de apoyo internos y externos ejercen sobre la forma en que los padres refugiados comprenden y responden a las necesidades postraumáticas de sus hijos en entornos desfavorecidos y de bajos recursos posteriores al reasentamiento.

## 1. Introduction

Children and adolescents make up a substantial share of the forcibly displaced population, and many face an elevated risk of PTSD and other post-trauma distress following war and displacement (Blackmore et al., 2020). For those living in resource-poor areas of low- and middle-income countries (LMICs), war-related trauma exposures typically co-occur with material deprivation and restricted access to basic services, which can impede recovery and erode coping resources posttrauma (e.g. Ermansons et al., 2023). When healthcare is inaccessible and children are separated from their wider support networks (e.g. peers, teachers), parental responses can become particularly critical as an immediate source of emotional support for children posttrauma (Betancourt et al., 2010; Sriskandarajah et al., 2015). However, when parents and children are co-exposed to war-related trauma, this shared relational context can undermine parents’ capacity to regulate their own emotional states and to co-regulate their children’s, increase maladaptive parental responses (e.g. harsh discipline, reactive parenting), and reduce parents’ responsiveness to their child’s post-traumatic needs (Relational PTSD; El-Khani et al., 2016; Scheeringa & Zeanah, 2001). This may, in turn, reduce trauma-related discussion and emotional support within the family at a milestone in resettlement when meeting children’s support needs is vital for integration and recovery (Kevers et al., 2024).

The association between parental trauma exposure and higher likelihood of increased harsh parenting, reduced warmth and responsiveness, disorganised attachment, and greater parental control has been documented across refugee and resettled caregiver samples (e.g. Bryant et al., 2018; Eltanamly et al., 2022; van Ee et al., 2016). Qualitative research has also revealed post-traumatic growth amongst refugee parents, which translated into increased parental warm engagement, compassion and leniency (e.g. Eltanamly et al., 2022). Evidence to date on the parental response to child’s trauma-related distress, however, is weighted either towards a set of homogeneous refugee parents (e.g. Syrian families in Jordan or Lebanon) or well-resourced resettlement settings in high-income countries (HICs). Of these studies, importantly, none interviewed refugee parents with a probable diagnosis of PTSD and a history of child trauma exposures, which can limit the clinical relevance of observations in the literature. Moreover, these findings may not be extrapolated to socio-economically disadvantaged and ethnically diverse refugee families in LMICs, where many live in impoverished conditions. For example, Eltanamly et al. (2022) drew on a relatively highly-educated and ethnically homogeneous sample in the Netherlands, and Sim et al. (2023) recruited government-assisted refugee parents in Canada who received government income and settlement support. As such, we know relatively little about how the intersection of war-related trauma and context-related factors may influence the way parents perceive and respond to their child’s posttrauma needs (Perzow et al., 2018).

The evidence gap relating to the experiences of refugee families in LMICs is critical, given growing recognition that material, social, and policy characteristics of the post-displacement ecology, specifically macro-level stressors, play a key role in refugee parenting through their effects on parental mental health (e.g. Ermansons et al., 2023; Khraisha et al., 2024). Qualitative research has shown that parental coping and responses cannot be decoupled from the social and economic contexts of the post-migration environment, and may be weakened or strengthened depending on the host country's resources (Khraisha et al., 2024; Miller & Rasmussen, 2017). For instance, across studies, housing restrictions that led to overcrowded living conditions among Somali and Palestinian refugee families, in turn, contributed to harsher, more controlling parenting (Ahmad & Smetana, 2021; Hickman et al., 2008), whereas Syrian refugee parents reported more communicative parenting after regaining stability following resettlement (Eltanamly et al., 2022). Despite these examples showing that socio-environmental factors are connected to parenting under displacement, the underlying pathways in which these interconnected post-resettlement stressors and supports co-constitute parental responses remain poorly understood.

Iran currently hosts more than 2.5 million Afghan, Iraqi, and Pakistani refugees, making it one of the world’s largest refugee-hosting countries. The influx of refugees from Afghanistan is particularly marked, comprising a population that has experienced decades of war, poverty, and severe restrictions on basic rights, including educational access. For example, an estimated 2.2 million girls are currently denied secondary schooling in Afghanistan, with high rates of illiteracy previously reported amongst resettled refugees in Iran (Abbasi-Shavazi et al., 2008). Iran itself is grappling with major economic and political challenges, with its refugee population correspondingly facing severe hardships. Despite the remarkably large and highly vulnerable refugee population in Iran, it is severely under-represented in the existing evidence base (Rajabi et al., 2026). Accordingly, we aimed to gain a nuanced understanding of parental responses to child’s post-resettlement support needs in the context of displacement and resource limitations in Iran. We focused on refugee parents living in resource-poor communities typical for Iran, where structural factors, such as chronic poverty and lack of access to basic services, intersect with parents’ own trauma histories and distress (Naseh et al., 2018). Specifically, this study explored: (1) how these refugee parents understand and interpret their child’s posttrauma distress; (2) their experiences of providing support to their child following trauma and displacement, including perceived barriers and available resources; and (3) how parents’ own trauma and displacement-related stressors influence *how* they understand children’s needs and their caregiving responses.

## 2. Methods

### 2.1. Participant recruitment and setting

Participants were 30 refugee parents resettled in Iran. Parents were recruited through an established partnership with the Association for Protection of Refugee Women and Children (HAMI), an international Non-Governmental Organisation (NGO) that delivers multimodal support to refugee families in resource-poor settings in Iran. Recruitment took place between April and November 2023 in three provinces, including Semnan, Mashhad, and Tehran. Alongside marginal settlements near Tehran, Mashhad and Semnan are both key hubs for refugee settlement, primarily due to job opportunities, low cost of living, and geographic proximity to Afghanistan and Pakistan. These cities accommodate refugees in formal and informal housing. Due to high unemployment rates and widespread poverty, the majority of these families live in socio-economically disadvantaged communities.

Parents were eligible to participate in the study if they: (i) were 18 years of age or older; (ii) had resettled in Iran as a refugee or asylum seeker; (iii) were the biological parent or primary caregiver of a refugee child aged 6–17 years; (iv) had capacity to understand and consent to study procedures; (v) had no known child protection concerns. The lower age limit of 6 years was selected because parenting responses and trauma-related distress are likely to present differently in very young children compared with older children and adolescents, as reflected in DSM-5’s separate PTSD criteria for children aged 6 years and younger.

Recruitment was conducted by HAMI research staff within local offices in refugee communities. The HAMI research team conducted individual and group outreach meetings with refugee parents to introduce the study, including parents from both informal tented settlements and formal housing. Parents who expressed interest in participating were asked for details and received the study information sheets. Due to potential issues around literacy amongst the refugee populations, HAMI researchers read the information sheet aloud to ensure all aspects of the study were clearly understood. HAMI staff subsequently contacted parents within 3–5 days to see whether they wished to take part and arrange a time for the assessment. All interested participants were given the opportunity to ask any questions related to the research and their participation before providing consent. HAMI research staff were Farsi speaking, but interpretation was provided as needed during recruitment and data collection for non-Farsi speaking participants.

Of the 84 potentially eligible refugee parents initially approached by HAMI researchers, 51 declined or did not attend the scheduled interview. Additionally, three participants requested to withdraw their data within three weeks of their participation. In total, 30 parents completed study assessments (see Figure S1 for the recruitment process in the Supplementary File). No additional participants were approached after recruiting 30 parents, as the primary researcher (MR) determined that further interviews were unlikely to generate new themes (Hennink & Kaiser, 2022).

### 2.2. Measures

#### 2.2.1 The Post-traumatic Diagnostic Scale-5 (PDS-5; Foa et al., 2016)

The PDS-5 is a 24-item adult self-report measure comprising a trauma history screen, followed by 20 items indexing PTSD symptom severity in the last month based on the diagnostic criteria outlined in DSM-5. Scores for the total PDS-5 range from 0 to 80, with scores of ≥28 suggesting a probable PTSD diagnosis (Habib & Zulfiqar, 2025). We followed the clinical guidelines used in a refugee sample to categorise symptom severity as follows: minimal (0–10), mild (11–23), moderate (24–42), severe (43–59), and very severe (60–80). The scale has shown good internal reliability and validity (alpha = .90) and has been used in refugee populations (e.g. Renner et al., 2021). In this study, PDS-5 scores were used descriptively to characterise parents’ current PTSD symptom burden and to contextualise qualitative accounts of parenting, distress, and caregiving capacity.

#### 2.2.2 UCLA PTSD Reaction Index trauma screen (UCLA-RI; Pynoos et al., 1998)

The parent-report version of the University of California at Los Angeles (UCLA) PTSD Reaction Index (UCLA-RI) captures children’s trauma exposure and PTSD symptoms. Part 1 of the parent-report version of the UCLA-RI was completed by participants in the current study, comprising a yes/no checklist of 13 possible trauma exposures, including war, natural disasters, physical or sexual abuse, serious accidents, and medical trauma. The resultant score is a count of events. This information was used descriptively to characterise the focal child’s trauma exposure and to contextualise parents’ interview accounts. Parents with multiple children in the study age range were asked to identify a single child to focus on throughout.

#### 2.2.3 Semi-structured interview

A semi-structured interview was developed for this study. The development of the interview guide was informed by the existing evidence on parental responses to child trauma in refugee families, as well as by a focus group with HAMI researchers to ensure cultural relevance and sensitivity. The initial version was piloted with two parents and identified issues (e.g. long or too complex probes), which were discussed in a second HAMI focus group and the interview guide was further refined. The final interview comprised demographic items followed by questions to explore: (i) parents’ perceptions of their child’s trauma-related reactions and the ways they responded to the child’s distress; (ii) the impact of parents’ own trauma on parenting; and (iii) parents’ experiences of post-resettlement stressors and structural constraints in Iran, and their influence on parents’ capacity to support their child (see Appendix S1 in Supplementary File for a detailed version of the interview guide).

The original version of the interview guide was developed in English prior to being translated to Farsi by the lead author (MR), who is bilingual. Subsequently, the Farsi version of the interview guide was back-translated into English by two bilingual translators in Iran. Where needed, HAMI researchers translated the interview guide to other languages for non-Farsi speaking participants.

### 2.3. Data collection procedures

Prior to data collection, the lead author (MR) provided six training sessions to HAMI researchers (*n* = 5, all female), including research ethics, consent procedure, qualitative interviewing methods, engaging with trauma-exposed individuals, debriefing, and risk and referral process. In addition, during data collection, MR was present in Iran to supervise a subset of interviews, providing detailed feedback on interview content through daily meetings, in order to ensure appropriate focus and prompting on relevant topics. Field notes were additionally recorded, capturing contextual observations. The emotional well-being of the HAMI researcher team was also regularly discussed, and support was provided where needed (see Table S1 for more details about the HAMI researchers in Supplementary File).

All interviews with parents were conducted in person at HAMI offices (Semnan = 21, Mashhad = 6, Tehran = 3), audio-recorded only, and later transcribed in Farsi by the lead author (MR) and HAMI staff. Demographic questions and the study questionnaires were verbally administered by HAMI researchers due to the low literacy levels of participants, followed by semi-structured interview questions. On average, interviews lasted 37 min (range: 22.3–73.1 min), and the full assessment process, including the completion of questionnaires, took no more than 90 min. Most interviews were conducted in Farsi, the preferred language of most participants, with the exception of two interviews that were conducted in Arabic (*n* = 1) and Urdu (*n* = 1) with support from local interpreters. Following participation, parents were thoroughly debriefed and compensated for their time (£8).

### 2.4. Data analysis

Qualitative data were analysed using inductive thematic analysis (Braun & Clarke, 2006). We selected this method because it is well suited to explore individuals’ experiences and perspectives, and has a systematic yet flexible framework for robust analysis of qualitative data (Braun & Clarke, 2022). We adopted a predominantly inductive approach for our data analysis, allowing patterns and meanings to emerge directly from the data, grounded in participants’ narratives. To avoid ‘common problems’ in the use of thematic analysis and strengthen methodological clarity and coherence in the analysis and reporting of data, we followed Braun and Clarke’s (2006), 15-point checklist for good thematic analysis. More information on our adherence to quality criteria can be found in Table S2 of the supplementary file, which maps this study to the COREQ checklist for reporting qualitative research.

We followed Braun and Clarke’s (2006, 2023) six-phase process for analysis. Two bilingual researchers (MR & FB) led the data analysis process. Stage 1 involved familiarisation with the dataset through listening to all recordings, reading transcripts, and reviewing the field notes. In stage 2, MR and FB systematically generated initial codes, based on reading and re-reading of transcripts, taking an inductive approach. In stage 3, MR and FB met and began clustering coded data into candidate themes. In stages 4 and 5, the initial codes and candidate themes were discussed with the research team and HAMI researchers, and were presented to a group of refugee parents by HAMI to support refinement. In the final stage, MR wrote up the finalised analysis, incorporating multiple rounds of review and feedback from the whole author team (see Appendix S2–S4 for the positionality statement, researchers’ background, and epistemological orientation in Supplementary File).

### 2.5. Ethical and safety considerations

Informed consent procedures emphasised confidentiality, participant right to withdraw, and the voluntary nature of participation, being independent of receipt of social support from HAMI or any governmental organisation. If assessments identified concerns relating to parental or family well-being, HAMI researchers followed standard risk protocols and made direct referrals to their clinical services where necessary. These services were readily available and free of charge for refugee minors, adults, and other family members of the participants. All safeguarding procedures and the study protocol were reviewed jointly by the research team and HAMI staff and were approved by the University of Bath Psychology Research Ethics Committee (PREC ID: 23-022).

### 2.6. Community feedback and validation of findings

To strengthen the credibility and methodological integrity of the analysis, a simplified version of the preliminary findings was presented to a small group of Afghan refugees, including research participants (*n* = 3) and members of the refugee community (*n* = 4), after the initial development of themes. Led by HAMI researchers, attendees were shown a brief and visual summary of the emerging themes and associated sub-themes. HAMI researchers invited the attendees to comment on whether the interpretations reflected their lived experiences in Iran, whether anything could be further highlighted or missing, and whether alternative meanings should be considered. This was performed as a sense check of the interpretations, as well as an opportunity to gain further socio-contextual insights into the data. During this session, attendees also discussed the potential policy and practice implications of the findings. Feedback from this session was documented in analytic memos and shared with the research team. This process helped ensure that the initial themes were well grounded in the data and that contextual elements of parents’ lived experiences were adequately represented in the emerging analysis.

## 3. Results

### 3.1. Sample characteristics

Participants were 30 refugee parents residing in three provinces of Iran, comprising 21 mothers (70%) and nine fathers, aged 25–59 years (M = 41.6, SD = 8.53), all biological parents. The majority of participants (83%) were illiterate, 80% were originally from Afghanistan, 56% were unemployed. The length of stay in Iran ranged from 1 to 54 months (median = 15) (see Table 1 for full demographic details). Supplementary Tables S3 and S4 provide details of child trauma exposures and PDS symptom scores for each participant, in order to contextualise the findings. All participants reported exposure to at least one trauma type (see Supplementary Table S5 for a summary of focal traumas and PDS-5 total scores for all participants). Mean parental PDS-5 scores were in the moderate to severe range (M = 39.7, SD = 7.98), with all parents reporting at least moderate levels of PTSD symptoms (range 26–61).

**Table 1.** Sample characteristics (*N* = 30).

Parent Characteristic	Statistic
Age in years, M (SD)	41.6 (8.53)
Proportion of mothers, *n* (%)	21 (70)
Country of origin, *n* (%)	
Afghanistan	24 (80)
Pakistan	4 (13.3)
Iraq	2 (6.7)
Educational level, *n* (%)	
Illiterate	25 (83.3)
Primary school	4 (13.3)
Secondary school	1 (3.3)
Proportion unemployed, *n* (%)	17 (56.7)
Resided in Iran (months), median (range)	15 (1–54)
Undocumented asylum seeker or refugee, *n* (%)	17 (56.7)
Number of children per household, M (range)	4.63 (2–10)
PTSD symptom severity scores (M, SD)	39.7 (7.98)
Number of trauma exposures, median (range)	2 (1–6)
**Child characteristics**	
Age in years, M (SD)	10.4 (2.93)
Proportion of girls, *n* (%)	17 (56.7)
Number of trauma exposures, median (range)	4.50 (1–9)

### 3.2. Qualitative findings

Inductive thematic analysis generated four themes relating to parents’ experiences in understanding and supporting their child’s needs as a refugee, as well as the displacement challenges they faced in resource-limited contexts. Four themes were developed, namely: (1) Parents’ experience in understanding and interpreting children’s distress; (2) Parenting under displacement: depleted resources and inherited practices; (3) Intra-family dynamics; and (4) Support needs, help-seeking, and care pathways. A summary of these main themes and their sub-themes is provided in Table S6 in the supplementary file.

#### 3.2.1 Theme one: parents’ experience in understanding and interpreting children’s distress

In this theme, refugee parents described how they perceived and interpreted their children’s emotions and needs in the aftermath of trauma, and how they understood their own trauma, beliefs, and ideologies as shaping responsiveness and daily parenting. The following sub-themes were identified: (1) Uncertainty about when and how to provide support, (2) Little understanding of mental health as a concept, (3) Support provided for children’s physical symptoms only at crisis point, and (4) Spiritual resilience: a resource for making sense and bouncing back. Next, we will describe each of these subthemes.

#### 3.2.2 Sub-theme one: uncertainty about when and how to provide support

When refugee parents were asked to explain their responses to changes in their child’s emotions and behaviours following highly distressing events, almost all parents reported that they did not know what to do or what is ‘right’ to do. Throughout interviews, considerable uncertainty about a proper response to the child's emotional needs was consistently described.

Interviewer: In those days, what did you do to make the child feel better?

Participant: We didn’t do anything. Now I think he is fine. One moment he is fine, one moment he is bad. It has become like this, my son. I didn’t know what I should do.

Interviewer: Now what? How do you help him?

Participant: Nothing. Now I don’t know. I think he is fine. (Mother of four children, age 44, Afghanistan)

A perceived inability to respond effectively to the trauma-related needs of children and the sense of having no control over their responses were also described by parents, with behavioural disengagement or a strong sense of helplessness being a fundamental feature of their coping mechanisms.

Interviewer: What were your children doing?

Participant: They were crying.

Interviewer: How about you?

Participant: I was crying too.

Interviewer: What did you do to make your children quiet and calm?

Participant: I didn’t know what to do with them. I didn’t do anything. I was just crying. (Mother of two children, age 25, Afghanistan)

A minority of parents (*n* = 2), educated compared to others, were more proactive in providing support in positive ways, such as offering reassurance and physically warm gestures (e.g. hugs) or being present to calm down their child.

Interviewer: When you arrived [in Iran], did you talk to them about what they got through in the journey?

Participant: Yes, I knew my child. I hugged them and sat with them to [help them] feel less scared. (Father of two children, age 54, Iraq)

#### 3.2.3 Sub-theme two: little understanding of mental health as a concept

Parental descriptions evidenced a significant lack of mental health literacy, with the complexities of psychological distress and mental illness not clearly parsed or well understood by refugee parents. Several parents struggled to understand questions about the well-being of their children, or they had only a very basic grasp of this concept. Many lacked words to describe more complicated or trauma-related emotions, states, and symptoms. As a result, parents found questions on their children’s well-being confusing and gave very broad, simplified answers, such as whether the child was ‘happy’ or ‘not feeling good’ posttrauma.

Interviewer: What do you think you could have done for the children to feel better, with these events, that you didn’t do?

Participant: I don’t know.

Interviewer: Did you do anything to make his crying or upset less?

Participant: Sorry, I don’t understand what you are asking. I don’t understand. (Father of four children, age 45, Afghanistan)

Refugee parents also struggled to distinguish emotional and psychological distress from physical health conditions. For example, in most cases when parents were asked how they would provide or seek support if their child felt emotionally unwell, they described that they would do something only when physical signs were present (e.g. a cold, fever, or headache).

Interviewer: In your opinion, when they get like this, do your children need counselling [or] help from a psychologist?

Participant: No, they don’t [need it]. If they get cold or something, I take them to the doctor. My daughter had a very bad headache, [so] I took her. (Mother of seven children, age 34, Afghanistan)

#### 3.2.4 Sub-theme three: support provided for children’s physical symptoms only at crisis point

According to parents, many refugee minors had experienced physical distress, injuries, and life-threatening events both in their home country and during the migration journey. Parents spoke of incidences that left children with physical impairments, and they reported noticing changes in the child’s behaviour soon after. However, they described very high thresholds for providing support or seeking formal services, including severe and acute symptoms that were clearly visible. Consequently, parents usually sought care for their children only for serious physical health symptoms, with routine appointments, monitoring, and early help-seeking commonly postponed, particularly when the child's symptoms fluctuated or did not appear immediately dangerous.

My son fainted for a whole day and night … then we took him to the hospital to get an image of his head. (Mother of four children, age 44, Afghanistan)

My twins have thalassemia[;] they get fever a lot and get sick. Unless we have to, we don’t take them to the doctor. (Mother of four children, age 36, Afghanistan)

In several cases where seeking healthcare was described as being postponed or not possible, refugee mothers narrated how they received intergenerational advice, particularly from elder family members, to use home remedies or basic practices to alleviate symptoms temporarily.

We had previously gotten something from the pharmacy. I also called my mother [to ask] what I should give her so her pain would be less. We tried, for now, to make her better ourselves. (Mother of three children, age 36, Afghanistan)

For refugee families who sought help for their child’s physical symptoms, once these had been addressed, they did not seek additional (non-physical) support for the child’s crying or withdrawal, even where the need for such support was indicated.

Interviewer: Did you take your child to counselling after that? Because he was crying a lot?

Participant: No. That day that I took him to the hospital, he got better. It was enough. (Mother of ten children, age 37, Afghanistan)

#### 3.2.5 Sub-theme four: spiritual resilience: a resource for meaning-making and recovery

A recurring pattern across interviews was how parents used spiritual beliefs to make sense of past trauma and manage ongoing hardships in deprived communities after arrival. Although parents occasionally spoke first about their own suffering, their narratives revealed a broader interpretive framework through which they understood their children’s emotional and physical difficulties. Parents often viewed war trauma and displacement-related difficulties as part of ‘God’s plan’, believing that God had prepared them to remain patient and strong in the face of adversity. In this way, spiritual beliefs made displacement-related suffering more understandable, helped them tolerate uncertainty about their children’s needs, supported them to make positive meaning from current hardships, and strengthened their belief that they could continue caring for their children despite limited resources. Faith-based practices, such as daily prayer, reading the Quran, and visiting Holy Shrines, were therefore not only coping strategies, but also part of how parents interpreted suffering, maintained hope, and understood their role in responding to their children’s distress.

I worry about what will happen to my children … I have been through a lot of hardship myself, but God helps, and these difficulties will pass too. At night, I pray for my children and ask God that they become successful and proud, [and that] I can support them. Imam Reza (peace be upon him) and Imam Hussein (peace be upon him) have helped us a lot. (Mother of ten children, age 50, Afghanistan)

Interviewer: OK, how did you resolve it? What did you do?

Participant: I put my trust in God. I pray and read the Quran, and say prayers … . I owe getting through all the problems to God, who helped [me]; and the more the problems increased, the more my faith in God increased. I truly got calm from God and I feel a lot of satisfaction. He gave me a lot of strength to pass. (Mother of five children, age 49, Afghanistan)

Although religious beliefs were a strong source of meaning and a driver to move forward posttrauma for most refugee parents, a minority also adopted a more passive stance. They attributed hardships to ‘God’s will’ and waited for his help, instead of taking action. This fatalistic coping/belief made them more reluctant to seek support.

Interviewer: In your opinion, what could you do to help [your] children feel better [in Iran]?

Participant: That is God’s decision … 

Interviewer: Did you get help from someone or somewhere for them, so these problems become less?

Participant: No, there was no need. (Mother of seven children, age 55, Pakistan)

#### 3.2.6 Theme two: parenting under displacement: depleted resources and inherited practices

The parents’ narratives revealed that displacement experiences did not just change posttrauma parenting responses; they also reshaped their overall capacity to provide care as a refugee parent. Participants exemplified the multifaceted factors that influenced their caregiving behaviours across the migration trajectory, highlighting the role of familial influences, physical consequences of trauma, and functional impairments within the post-migration environment. This theme comprised three sub-themes: (1) Parenting is learnt intergenerationally, not taught, (2) Chronic pain and health problems impair caregiving ability, and (3) When post-resettlement stressors provoke harsh parenting. Next, we will describe each of these subthemes.

#### 3.2.7 Sub-theme one: parenting is learnt intergenerationally, not taught

Overall, refugee parents evidenced limited capacity for reflection on their own parenting. A lack of in-depth reflection on how their reactions or parenting behaviours might influence their child’s inner experiences (i.e. thoughts, emotions) was coupled with mothers in particular reporting that they followed, largely unquestioningly, their sisters’, mothers’, or grandmothers’ parenting advice, even when they did not know the rationale behind those practices. Low literacy and lack of community services in the host community were factors that reduced opportunities for parents to learn alternative responses or seek guidance beyond intergenerational family relations.

Interviewer: Did you get help from anyone on how to help your son [after restatement]?

*Participant:* No; well, my mum always taught me. For example, she said I should feed [them] and raise them. I didn’t have anyone [in Iran]. (Mother of three children, age 27, Afghanistan)

Many refugee parents described frustration at their inability to provide routine support to their child in the same way as before displacement, given the overwhelming demands of resettlement. They believed that meeting basic needs of children (e.g. feeding) should be sufficient after displacement, and showed awareness of but limited insight into why parent–child conflict had escalated. Aside from a small number of traditional responses (such as arranging marriage for girls, or child labour for boys as a way to manage arguments), parents did not describe any initiatives or coping methods to address increased parenting challenges and the child’s emerging needs in Iran. Instead, with one exception, when asked whether their parenting approaches needed to change after displacement, parents emphasised that they continued to rely on previous familial traditions and resources.

Participant: I didn’t change [my behaviours], as we are illiterate.

Interviewer: Just like how you treated them in Afghanistan, you treated them the same in Iran?

Participant: Yes … what could I do? They bother [me] a lot. I don’t have the energy here either. (Mother of three children, age 55, Afghanistan)

During discussions about parenting after displacement, refugee mothers often described fathers as having markedly low engagement in everyday caregiving, much like their own fathers, being ‘quiet’ or even ‘inattentive’.

My husband is quiet. He generally doesn’t do anything [at home], and he doesn’t pick on my child much. (Mother of four children, age 36, Afghanistan)

#### 3.2.8 Sub-theme two: chronic pain and health problems impair caregiving ability

Many refugee parents highlighted that caring for their children was physically and emotionally demanding, especially in the first months after resettlement. Several parents were physically injured or reported acute and long-term physical challenges, such as cardiovascular diseases, recurrent ‘headaches and fatigue’, ‘dizziness’, ‘stomach pain’, and sleep problems. Parents linked these chronic physical symptoms with reduced capacity to provide adequate care for their family, including managing domestic tasks, taking the child to appointments, following up their child’s schoolwork, as well as carrying out other routine tasks and employment.

Yes, when we were in the Mashhad camp, everyone was sick. I was sick too. I had a uterine prolapse. I suffered a lot. I [also] have gallbladder disease and a stomach problem. Well, I was suffering a lot because of illness. [So] I couldn’t do household chores [and] children’s work. (Mother of four children, age 44, Afghanistan)

For some parents, physical health conditions were described as so severe that parents felt unable to remain emotionally responsive and available to their child. In addition to the emotional burden of trauma and processing the grief of losing a child or husband during migration, they described ongoing pain and functional limitations as major barriers to spending quality time with their child or responding to their needs.

Participant: For a long time after I arrived, I had severe heart racing [palpitations]. When it [those days] comes back to my mind, I suddenly get very stressed. I also have [high] blood pressure with dizziness.

Interviewer: Did this impact your behaviour with the children?

Participant: Yes. When you are sick, you can’t fully look after your children [or] take them out to play … I'm not in the mood anymore. (Mother of eight children, age 45, Afghanistan)

Being overly sensitive was a persistent feature amongst refugee parents struggling with trauma-induced injuries and chronic pain, with some parents describing becoming easily irritated or angered by everyday noise in the home, such as their children laughing or shouting.

Interviewer: And now, do you think these illnesses affect your children?

Participant: Yes, it affects [them]. As soon as they make noise, my nerves get upset. (Mother of two children, age 59, Afghanistan)

#### 3.2.9 Sub-theme three: when post-resettlement stressors provoke harsh parenting

While we did not directly ask parents to compare their parenting before and after displacement, many retrospectively described their routine caregiving in their home countries as supportive and responsive when children felt unsafe, for example, due to war or regional conflict, with only a few parents (*n* = 3) reporting a history of harsh discipline. Parents gave examples of holding their child close and offering reassurance when the child experienced horrific events pre-migration (for example, the shooting of friends in the community, the sudden death of relatives), whilst encouraging their child to stay vigilant.

Interviewer: At that time when your children were scared of the Taliban, what did you do?

Participant: When I was in Afghanistan, I would bring something for him so he wouldn’t be scared. I would calm him. I would say we shouldn’t go outside, it’s not safe. (Mother of three children, age 36, Afghanistan)

During displacement, all refugee parents had been exposed to profound adversities and war-related trauma. After resettling in deprived areas in Iran, they faced additional stressors, including housing difficulties, financial pressure and high living costs, malnutrition, low-paid work, barriers to enrolling their children in local schools, and experiences of discrimination/abuse from the host community. Parents’ narratives suggested that war exposure, together with ongoing resettlement stressors, was linked with heightened emotional reactivity and harsher or more punitive responses towards their children. In contrast to their pre-migration narratives, many parents described how daily post-resettlement stressors – rather than past war traumas – provoked them to lose their temper. They felt unable to tolerate their children’s demands and described their frustration, which led to physical punishment or deprivation. Some fathers also reported using similarly harsh practices towards their partners following resettlement.

Sometimes it is the pressures of life; I start [too much] overthinking things. I can tolerate it up to a point … I do not have work here to earn money, nothing. Most of the time, when we have no money here, or when the life became hard, I hit the them [children]. (Mother of seven children, age 34, Afghanistan)

I wasn’t like this before. Now, when they [my children] make too much noise, I would hit them. I’m fed up at home. (Mother of ten children, age 37, Afghanistan)

Interviewer: When they were crying a lot and you didn’t understand their pain, how did you calm them?

Participant: Nothing, or I would hit him … or, I don’t know … I had to [hit him], what could I do? I would get tired. We had nothing. There wasn’t a doctor to relieve his stomach pain. (Father of four children, age 45, Afghanistan)

Across the sample, only two refugee mothers explicitly connected their war-related symptoms with feelings of anger and described how this sometimes contributed to harsh parenting practices. Housing difficulties and cramped living conditions were also identified as important factors that intensified these responses.

Participant: Because I don’t have the nerves for it [making noise] either, and it keeps affecting my neck and my stomach.

Interviewer: Oh, after that attack [by the Taliban that] you said?

Participant: Yes. Well, when they make noise at home, my nerves get messed up, and I feel like I want to throw them out or hit them. Our home here is small, and we can’t go outside … It doesn’t have a yard. (Mother of six children, age 36, Afghanistan)

#### 3.2.10 Theme three: intra-family dynamics

The key focus of this theme was how repeated displacement-related stressors, deprivation, and economic hardship in the post-resettlement context impacted the relational processes of family caregiving, particularly through their influence on routine parental responses, parent–child communication, and daily interactions in the household. This theme comprised two key sub-themes: (1) Communication gaps in understanding children’s support needs, and (2) Children take on parenting-like responsibilities within the family, described below.

#### 3.2.11 Sub-theme one: communication gaps in understanding children’s support needs

Conversation about children’s adverse experiences or distress was described as limited, with refugee parents highlighting that their children rarely shared their negative feelings, thoughts, or physical signs of what they had been through. Most children were described as keeping personal stories to themselves, and parents did not attempt or described lacking effective strategies to encourage child disclosure. Parents often reportedly assumed that if the child was not talking about their feelings, this silence meant they were ‘fine’, with no further follow-up sought.

Interviewer: Do your children share their feelings with you?

Participant: No.

Interviewer: Do you talk [with them]? How about with your daughter? Does she share her feelings with you?

Participant: My daughter and my son are [both] fine. There’s no need for them to say anything. (Father of four children, age 45, Afghanistan)

One possible reason for poor communication about trauma-related incidents was a wish for mutual protection in the family. Parents described various occasions on which they realised their children were keeping upsetting stories or severe symptoms to themselves to avoid compounding their parents’ existing challenges.

His hand was injured several times [at work]. I realised he had not told me because he did not want us to spend money on a doctor. (Father of four children, age 45, Afghanistan)

Later, my son said, “Mum, I didn’t say it because you are sick, so your condition wouldn’t get worse.” He hadn’t told us anything. (Mother of four children, age 44, Afghanistan)

Refugee parents also did not explicitly speak about their own lived experiences of war, chronic pain, and displacement difficulties with each other within the family. This silence was used to protect their younger children and spouses, and for some mothers, to avoid triggering distressing emotions. Some parents disclosed their trauma-related stories for the first time during this study.

Interviewer: You didn’t say anything?

Participant: No. So that the children wouldn’t get upset, I didn’t say anything, whether I have [physical] pain or I don’t. (Mother of seven children, age 55, Pakistan)

#### 3.2.12 Sub-theme two: children take on parenting-like responsibilities within the family

As a result of ongoing challenges of precarity and material poverty in the post-resettlement environment, most refugee fathers had been unemployed or unable to provide consistent care and meet the financial needs of their families. Some also needed a carer due to severe war-related physical impairments. This meant children often undertook parenting-like responsibilities across practical and emotional domains to meet family needs. Many children had to work in age-inappropriate, harsh environments (e.g. brick kilns) to meet the basic financial needs of their family. While this child financial contribution was viewed positively by parents as a supplementary source of income, they also recognised negative consequences, describing limited access to education due to long working hours, extreme fatigue, and physical symptoms as some of these negative outcomes.

Their father has had a stroke and [is] now disabled. But my son works. He gets 8 million [£54] a month, [and] we spend [it] on food and eating … My son has become discouraged from going to school. He would get very tired. (Mother of five children, age 49, Afghanistan)

Children were also described as providing emotional support to their parents and younger siblings in stressful situations. Most parents shared examples of children trying to calm family members, ‘look after’ siblings, or stay close when distress escalated – both after severe adverse events and during ongoing displacement-related challenges.

No, I had a lot of pain in the beginning. My husband was martyred (passed away). My head was hurting, I couldn’t go to the doctor. My son said, “Come, I will take you to the doctor.” He always took me to the doctor and looked after me … Yes, [at that time] he was with me a lot. He was kind. (Mother of seven children, age 55, Pakistan)

#### 3.2.13 Theme four: support needs, help-seeking, and care pathways

When parents were asked about who they had sought support from, what resources they used, and what ideal support looked like for them, they repeatedly highlighted that daily stressors and poor living conditions had kept them in a protracted state of liminality, and meeting survival needs rather than providing proactive support for children’s emotional and developmental needs. We identified three sub-themes: (1): Meeting children’s basic needs outranks all other support needs, (2) Protective role of informal support networks, and (3) Accessibility, affordability, and helpfulness of psychosocial services.

#### 3.2.14 Sub-theme one: meeting children’s basic needs outranks all other support needs

When asked how they could be supported to respond to their children’s emotional distress or behavioural changes post-displacement, both fathers and mothers stressed that monetary support to help them meet their child’s daily needs was their highest priority, and a prerequisite for any other form of support. Most families were struggling to secure safe housing, pay rent, buy food, and access essential healthcare, and non-financial, psychosocial forms of support were not identified as a ‘need’ against this backdrop. When their basic needs were met, refugee parents typically felt ‘satisfied’ with their parenting and responses to the child.

Interviewer: After these hardships your children have seen, what help do you think you or your husband needs [to make your children feel better]?

Participant: The most important help is just food and these things. (Mother of three children, age 34, Afghanistan)

As long as there’s at least some bread … to give them [to my child], I get satisfied. (Father of two children, age 44, Pakistan)

Although interviewers tried to simplify the idea of psychosocial support after a child’s stressful experiences (by explaining counselling sessions, educational workshops, and parenting programmes as ways to better understand their child’s needs), most participants reiterated that their first priority after resettlement was still to meet their child’s basic needs, such as food and clothing.

Interviewer: What do you think your children need more that you do, to help them? For example, things that you can learn to understand their mood and feelings, or talk to them more.

Participant: Some food or something … We don’t have anything at home. If there is food, it’s better. If there is money, it’s better. Just [something like] that. (Mother of four children, age 37, Afghanistan)

Parents described that their difficult financial circumstances led them to overlook their child’s socioemotional needs, even when they identified support that could make their child happier. In some cases they described knowing what their child needed and finding it agonising that they lacked the resources to respond. This sense of helplessness had a profound negative effect on their own well-being, raising questions about whether they were a good parent.

Participant: He likes it when I take him to the park so he feels better, to play with him. I really like it too. My heart opens up [I feel better].

Interviewer: Did you take him?

Participant: No, we can’t afford the costs. What can I do [?] Our situation is like this … I feel bad that I can’t. (Father of four children, age 35, Iraq)

#### 3.2.15 Sub-theme two: protective role of informal support networks

Discussions about refugee parents’ support networks post-resettlement covered both formal supports (such as local charities, NGOs) and informal supports (e.g. relatives, community ties). Despite this variation, the underlying tone around perceived social support was consistent: any type of support received ‘immediately’ after arrival was highly valued, helping parents adapt to a new environment, navigate the Iranian health and educational system, and secure basic needs and schooling for their children in settings with limited resources and overstretched services. Although most parents were experiencing poor mental health post-migration, the support they described receiving from refugee-background relatives was, unsurprisingly, more instrumental (e.g. temporary accommodation, cash or food assistance) and informational (e.g. guidance on school registration), which mirrored refugee parents’ predominant needs in the early months after arrival.

In these one and a half years, for three months we were at my husband’s sister’s place and they were paying our expenses … they would guide us. About the children, school, medicine and the doctor. After three months, we borrowed money from relatives and acquaintances … and they helped so we could get this house. (Mother of four children, age 36, Afghanistan)

Amongst the different types of social support that positively influenced how refugee parents responded to their child’s needs, support from school teachers was mentioned most frequently. Almost all parents with school-aged children had at least one contact with their child’s teacher or school staff at the time of enrolment, which opened a communication channel to share their worries and discuss available school support and external services (e.g. referral to psychological services). Most refugee parents appreciated the combination of social and educational support provided by teachers. Notably, the positive impact of school-based support was especially clear for parents who reported exclusion by the host community, including experiences of racism.

Participant: Yes. Ms. Shojaei [the child’s teacher at school] is also a very good and kind woman.

Interviewer: How did she help you?

Participant: For registering the children. And in early days, [advice on] what to do with the children’s stomach aches and our problems here. (Mother of three children, age 34, Afghanistan)

#### 3.2.16 Sub-theme three: accessibility, affordability, and helpfulness of psychosocial services

Almost no refugee parents had used clinical, non-clinical, or community-based humanitarian mental health or psychosocial support services at any point in their migration process. Some mothers were manifestly distressed and described being overwhelmed by their traumatic experiences as refugees, yet concluded that they did not need mental health support to recover. Both mothers and fathers also reported that financial hardship prevented them from attending healthcare centres regularly, or that they felt their physical symptoms and associated care were in hand and thus they did not require other forms of care (e.g. psychological services), reflecting the common tendency to somatise emotional distress in the sample.

Interviewer: Do you think that after you came from Afghanistan, it is necessary for a counsellor or specialist to talk more with your children? For example, that you have a psychologist and they talk with the children.

Participant: My son has some problem[s]. He has a heart problem. But I did tests myself, I didn’t have a problem. (Mother of four children, age 37, Afghanistan)

When we asked parents how they dealt with their child’s emotional and behavioural changes after resettlement (for example, irritability or anger outbursts, sleep problems, or frequent crying), most either did not believe that the child had a problem needing professional support, felt they could manage it at home, or expected it would improve over time without specialist support.

Participant: It’s everyday life, there are problems and we have to solve them ourselves. I never thought that we need [it]. (Mother of four children, age 53, Afghanistan)

While many refugee parents were unaware of such support services, their younger children sometimes facilitated or encouraged access to mental healthcare. For example, one refugee father explained that his child encouraged him to seek help from specialised services so that he could talk to someone about his stress.

Interviewer: Did you go by yourself, or did someone suggest it to you?

Participant: My children had said, “You go to a psychologist and listen to what they say too, so your nerves can be a bit calmer.” (Father of five children, age 35, Afghanistan)

Many parents perceived their distress as an inevitable consequence of displacement-related stressors and questioned the effectiveness of discussing these issues with counsellors, believing their distress would be addressed only if their basic needs were met. Therefore, parents perceived little benefit in mental health services unless their housing, food, and income were secured first.

Interviewer: Do you think counselling would help you if you went?

Participant: No, these don’t help at all … Suppose I go to a counsellor. What can they do for me as long as I don't have a bundle of money? (Father of four children, age 37, Afghanistan)

## 4. Discussion

**Figure 1.**
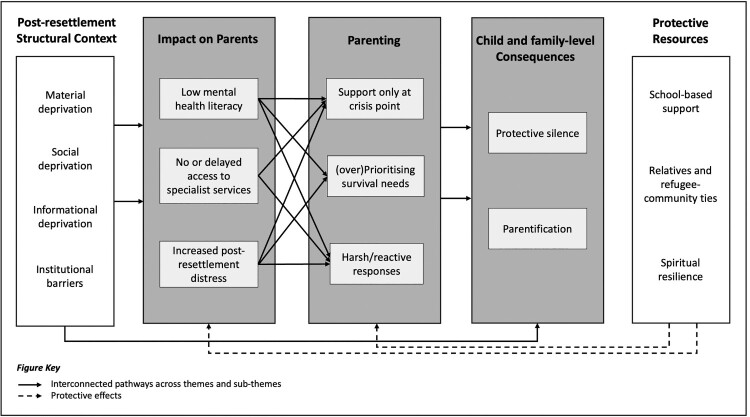
Concept map of the contextual factors influencing parental responses to the child’s posttrauma needs amongst refugee families in resource-poor settings.

This study aimed to explore how refugee parents exposed to war- and displacement-related stressors understand and respond to their children’s posttrauma distress and their experiences of support and coping in conditions of multidimensional deprivation in resource-poor settings in Iran. To this end, we interviewed Afghan, Pakistani, and Iraqi refugee parents living in socio-economically deprived communities, generating empirical evidence from a historically under-researched population. Four key themes were identified: parents’ understanding and interpretation of children’s distress; parenting under displacement; intra-family dynamics; and support needs, help-seeking, and care pathways. Our findings showed that, in the resource-poor communities studied, multiple forms of deprivation created a complex grid of social and economic determinants through which poverty, limited service access, low mental health literacy and restricted support resources interactively constituted parental responses to children’s posttrauma needs in refugee families (See Figure 1 for interrelated pathways identified in this study). The resultant insights can inform the adaptation of family and parenting interventions and provide policy blueprints that address macro-level challenges for conflict-affected refugee populations in Iran and other similar resource-limited contexts.

Qualitative analysis of caregivers’ accounts of caring for their children posttrauma identified themes relating to caregivers’ perceptions of their children's coping, strategies used to support their children, the impact of the event on the caregiver, experiences of support, and coping strategies employed. The results detail the major challenges faced by caregivers in supporting their children following trauma exposure in a high-adversity context. Children experienced significant distress posttrauma, and caregivers attempted to support their children with strategies that were, at times, not entirely coherent. Given the numerous barriers to psychological treatment, caregivers often struggled to access treatment for their children and felt anxious and unable to adequately care for or protect their children posttrauma. Caregivers’ involvement in and understanding of their children's psychological treatment was limited. Finally, caregivers’ own distress could be a barrier to providing support. These findings may have implications for family-based interventions posttrauma.

A major theme emerging from the data was related to refugee parents’ understanding of children’s posttrauma responses. While most participants described profound changes in their children’s behaviours and emotions following their experiences, no parent described their child’s distress with reference to trauma, PTSD, or any mental health condition. While this broadly aligns with prior work indicating limitations to caregiver understanding of trauma-related symptoms in both refugee and non-refugee samples (e.g. Christie et al., 2020; Slewa-Younan et al., 2014), this lack of understanding was particularly profound in our sample. The parents we interviewed lacked even a basic understanding of the emotional consequences of trauma and a conventional emotional terminology to describe children's feelings, which appeared to contribute to parental helplessness and inability to respond (Ehlers & Clark, 2000).

Our findings appear to contradict evidence from refugee caregivers in resource-rich environments, where Afghan and Iraqi refugees were able to identify vignette symptoms as related to trauma or PTSD (May et al., 2014; Slewa-Younan et al., 2014; Yaser et al., 2016), and reported some adaptive coping strategies, such as positive reframing and planning (Sim et al., 2023). In understanding this discrepancy, it is critical to consider the specific contextual circumstances of refugees living under conditions of chronic deprivation in LMICs. In such contexts, support for acculturation and adjustment is typically scarce. Prior studies suggest that resettlement in enriching environments, with greater exposure to informational resources (e.g. routine consultations with health professionals, raising awareness programmes), can help refugees to develop a better understanding of their family’s mental health needs and favourably influence attitudes towards help-seeking (Ermansons et al., 2023; Glorius et al., 2020; Siddiq et al., 2023). The contextual void in the current sample was further evident in parental responses when asked about support for their child's distress, where they identified meeting their child’s basic physical needs versus possible psychosocial responses or services. Parents were strikingly focused on physical or somatic symptoms throughout, even when asked directly about emotional distress. The comparatively lower socio-economic status and educational attainment of parents in our sample contrasts with studies in HIC resettlement contexts which have often recruited refugee parents with more educated backgrounds, a quality that aligns with higher parental reflective functioning and more elaborated understanding of children’s emotional needs (Eltanamly et al., 2022; Sim et al., 2023). Notably, the majority of parents in the current sample were illiterate and came from Afghanistan, a country with severe restrictions to educational resources; these factors may have further limited their mental health literacy.

A second theme was related to how combination of experience of war trauma and extreme deprivation adversely affected interrelated processes within the family. Most parents retrospectively highlighted supportive parenting responses, such as warmth and physically warm gestures, when reflecting on their pre-migratory parenting, despite challenges presented by war and violence. However, in the post-resettlement context, parents’ vulnerability to ongoing stressors and their inability to meet basic family needs heightened frustration, anger, and emotional reactivity. Most parents projected their anger towards their children through screaming, yelling, and physical punishment, reinforcing assertions that poverty-induced hardship in the post-resettlement context may exert a more powerful influence on family interactions than war trauma (Meyer et al., 2019; Miller & Rasmussen, 2017). This finding is also in line with qualitative work with non-displaced Afghan caregivers exposed to protracted war, where chronic poverty, accommodation difficulties, and social inequality had a greater impact on violent behaviour at home than the direct effects of war (Eggerman & Panter-Brick, 2010). Unfortunately, there remains a critical evidence gap regarding the mental health benefits of interventions that target daily stressors in resource-poor settings. Programmatic responses to prevent violence in refugee families should prioritise reduction of socio-economic risks associated with post-resettlement stress, alongside strengthening parent–child relationships.

A notable adversity-linked impact on parent–child interactions was reflected in children’s trauma communication and changes in family roles. Children were described as withholding information about the emotional or physical consequences of trauma to prevent parental distress and avoid household costs (e.g. unaffordable injury-related care). Other research has identified that child trauma disclosure can be influenced by child-level, family relational processes, and cultural variations (Dalgaard & Montgomery, 2015; Kevers et al., 2024; Sloover et al., 2024). Our study further highlights the role of the child’s macro-environmental context, specifically family economic hardship and wider duress, making child silence a multilayered, intentional response that could be negotiated by socio-economic factors. This protective approach also appeared in children’s adoption of parental roles, as children became ‘functioning adults’ who secured family survival needs when poverty and PTSD symptoms compromised parents’ capacity after resettlement, but were consequently exposed to additional harms (Chase, 1999). Perceived child avoidance of trauma-related talk was compounded by parental lack of exploration of children’s well-being, with silence being taken to indicate that a child was ‘fine’, despite clear evidence to the contrary. Future studies should recruit parent–child dyads or otherwise capture the voices of refugee children in low-resource communities to unpack further the mechanisms shaping trauma communication and parentification (i.e. a role reversal process in which children assume developmentally inappropriate emotional or caregiving responsibilities typically expected of parents) in refugee families (Hooper, 2007).

Finally, consistent with previous research, material and social deprivation in refugee parents’ environments shaped the hierarchy of their support needs and their capability to respond to them (Williams, 2008). In the current sample it was particularly evident that parents struggled to think beyond meeting children’s basic needs. Where parental ability to provide basic elements of physical care was compromised, parents described a loss of power in their parenting roles, harsher responses, with a consequent erosion of parental self-efficacy and doubts about being a good parent. Research examining the parenting experiences of Afghan and Pakistani refugees resettled in HICs found that when sufficiently resourced, parents reported a sense of relief at shifting away from basic concerns towards the socioemotional flourishing of their children (Rosenberg et al., 2022). Our study emphasises that place-specific social determinants of post-migration context can yield divergent parenting trajectories, such that some parents mobilise contextual resources in a gain-spiral with more positive outcomes, whereas others experience accumulating daily stressors with a downward spiral (Hobfoll, 1989). Notably, improving children’s well-being is likely to alleviate parental stress despite ongoing contextual challenges, since parental challenges and failures become an additional stressor. Nonetheless, for families resettled in Iran, psychological support for parents seems unlikely to be effective unless adapted to address co-occurring stressors and a fundamental lack of parental insight into the drivers and manifestations of children’s adjustment.

### 4.1. Study limitations

Our study has several limitations. We purposively selected participants which may limit generalisability. The high refusal and dropout rates may also limit the representativeness and transferability of the findings. Parents with stronger NGO connections or greater capacity to discuss sensitive experiences may have been overrepresented. This should be considered when interpreting the findings. All study parents had at least moderate PTSD symptoms, which potentially limited our capacity to parse findings according to adjustment, although it is notable that parents were not recruited on that basis. Our assessment of parental support for children was based on parental perspectives only, gaining child perspectives directly is critical to providing a full picture of their experiences. Finally, all interviews were conducted by local researchers from an internationally recognised NGO. Although interviewers were trained and supervised in qualitative interviewing, they were not highly experienced. Some interviews were challenging due to low participant literacy, leading interviewers to place greater emphasis on some prompts or to ask closed questions where participants were struggling to respond. A further limitation was related to the use of interpreters and translated interview data. Although local translators were used to minimise translation-related errors, their involvement and the subsequent analysis of translated transcripts may have increased the risk that some meanings were lost, altered, or misconstrued during the interview process.

### 4.2. Implications and policy recommendations

Findings from the current study have important applied implications. First, although mental health interventions have shown potential to improve refugee caregivers’ mental health (e.g. Narrative Exposure Therapy), our own and other emerging evidence from refugees in LMICs suggests that when profound post-migration stressors remain unaddressed, context-specific structural risks may continue to have direct and indirect effects on parenting. Addressing these is likely to lead to more sustainable improvements in family relationships and child psychosocial outcomes in resource-poor settings. Notably, in the current population mental health literacy was considerably low that it was essentially not recognised as a need by families, despite high levels of distress. Overcoming this barrier may require creative ways of engaging with families, such as embedding psychological care in the treatment of physical symptoms or provision of other support, including school-based networks that can identify needs and refer families to care (Rajabi et al., 2025; Akbari Zardkhaneh et al., 2023). To date, many trauma interventions are based on research in HICs, with adaptation for LMICS being mainly surface-level (e.g. language and terminology) (Ennis et al., 2020). Our research highlights that even research with equivalent refugee populations resettled in HICs likely fails to capture the fundamental conditions grounded in economic, cultural, and socio-political variables that constitute family recovery for families resettled in Iran and other resource-poor contexts. Given this, we call on interventionists to undertake substantive, deep-level cultural adaptations to the form, content and delivery of interventions, preserving fundamental functions while ensuring that the key contextual drivers relevant to the host population are meaningfully addressed.

## Supplementary Material

Supplementary File_28226_R1.docx

## Data Availability

The data that support the findings of this study are not publicly available due to the inclusion of information that could compromise participant privacy but are available from the corresponding author on reasonable request.
