# Supplementary material for: Post-resettlement context matters: a qualitative study of refugee parents’ responses to child trauma in low-resource settings
Source: Eur J Psychotraumatol. 2026 Jul 21;17(1):2693362. doi: 10.1080/20008066.2026.2693362 (PMC13390166; doi:10.1080/20008066.2026.2693362)

**Supplementary File**

Table of Contents

Interview guide 2

Researchers’ background3

Positionality statement4

Ontological and epistemological orientation 5

Details of HAMI researchers7

COREQ criteria for reporting qualitative research8

Summary of trauma types experienced as measured by the PDS-512

Summary of trauma types experienced reported by UCLA-RI12

Summary of focal traumas and PTSD symptom scores14

Themes and subthemes following thematic analysis17

Flowchart of the participant recruitment process 18

**Appendix S1. Interview guide**

| **Topic 1: Pre-migration life experiences** |
| --- |

Can you tell me about your life in your home country?

- Prior to the incident that made you leave <*HOME COUNTRY*>, how was your life?
- What was it that made you decide you had to leave <*HOME COUNTRY*>?
- When you left <*HOME COUNTRY*>, who came with you and how did you come?

| **Topic 2: Post-migration period and adaptation stage** |
| --- |

Can you tell me about your experience of coming to Iran?

- What were your hopes for coming to Iran?
- What happened to your family when you arrived?
- Were there any experiences that were particularly hard for your family when you first arrived? How did those affect you?
- Did you get any support? What was that like?
- How is life for your family now?

| **Topic 3: Emotional impacts of being a refugee** |
| --- |

- What impact has being a refugee had on your child(ren)?
- Do you think your own experiences in <*HOME COUNTRY*> have changed how you are with your children?
- How about since arriving in Iran, have your experiences as a refugee here influenced you as a parent?
- Are there any bad things that have happened to your children as a result of being a refugee here?
- How does that affect them? And what was your reaction to such distressing events?
- Do your children have any problems with their emotions or behaviours now? [*If yes:*]
- What do you think is causing those?
- How do you cope as a parent?

| **Topic 4: Mental health needs and available/ideal ways of support** |
| --- |

- Now I want to ask you about how you can support your children with their experiences.
- Do you feel your children need extra support to help them with their emotions or behaviours, following the experiences that made you leave <*HOME COUNTRY*>, or the challenges of being a refugee in Iran [*Why/why not?*]
- Is there anything that you have been doing to support your children with their experiences?
  [*If yes:*] Do you think this has been effective?
- Do you talk to your children about the things that have happened to your family as refugees?
  [*If yes:*] What kind of things do you talk about?
- Are there any barriers in your community that stop you from giving the support you would ideally like to your children? Or any things that make things easier for you as a parent?
- Looking back, is there anything you would have done differently?
- From your perspective, what is an effective/ideal way for parents to help their children cope with the challenges they face as refugees?
- Have your children had support outside of the family to help them with their upsetting experiences as refugees? For example, are there other family members, friends, religious groups, schools, or charities that have helped them to cope?
- Can you tell me what that was like?
- Have you experienced any racial discrimination in this process?
- What types of support or helping tools do you think are needed for families like you?
- Have you ever received any kinds of formal psychological services to support your child's wellbeing in Iran? [*for those who have responded yes:*]
- What was the treatment like?
- Who recommended to seek help from psychological services?

Appendix S2. Researchers’ background

The multidisciplinary and culturally diverse research team working on this project comprised researchers with backgrounds in Psychology and Clinical Psychology (MR, VW, RC, AK, SLH), Refugee Studies in the Middle East (FA), and Infancy and Early Childhood Development (FB). The research team had a variety of professional expertise and personal experiences from work with trauma‑exposed populations in high‑adversity contexts in LMICs and with displaced individuals in resource-poor settings. MR and FA previously taught and worked in Iranian schools, supporting marginalised and refugee children and adolescents from Afghanistan. FA is also the CEO of the HAMI NGO in Iran and has extensive experience in policy, research, and practice that support refugee families in Iran and Afghanistan. FB received training on children exposed to trauma, disaster and war, and MR, a doctoral researcher studying trauma-exposed children from refugee backgrounds, had experience in conducting research with the primary social networks of trauma-exposed children and adolescents in under‑resourced settings. In sum, the core research team combined professional and personal experiences in the field of PTSD and trauma, refugee studies, and mental health in high-adversity communities to lead this study and data analysis.

Appendix S3. Positionality statement

Throughout the study, the research team engaged with regular reflexive discussions to critically examine how their prior experiences, standpoints, and positionalities, especially during joint coding checking meetings between MR and FB, influenced the interpretation of the results. The team drew on the ecological model of psychological trauma (Harvey, 1996) and the ecological model of refugee distress (Miller & Rasmussen, 2017) to inform the analysis of how refugee parents responded to their children’s post-trauma needs in resource-poor educational settings. The study was based on the premise that, in LMIC resettlement contexts, refugee children often rely on informal sources of support, especially primary caregivers, and that post-resettlement living environment and macro-level stressors may play a crucial role in the quality of support caregivers can provide.

The authors actively reflected on how their own experiences, values, knowledge, and identities shaped the study. This included prior experience of conducting research on child mental health and trauma (MR, VW, AK, RC, SLH), experience related to refugee parents’ mental health and work with refugee families (MR, RC, VW, FA, SLH), and policy-level activities with refugee studies in Iran (FA). Reflexivity was achieved through group supervisory meetings and consultation meetings with HAMI for the duration of the project, as well as a reflexive journal that the lead researcher (MR) used in the course of this study to document assumptions, decisions, field notes, and evolving interpretations. These reflexive notes also documented contextual factors that shaped the interview process, including that most participants had very limited formal education and low literacy levels. In some cases, this affected how participants reported basic demographic information. For example, during the interviews when HAMI researchers asked one participant, “How old are you?”, the participant replied, “I don’t know… maybe 35 or 40” (PID 021). Such examples were treated not as inaccuracies in participants’ accounts, but as reflections of the broader structural conditions in which many refugee families were living, including disrupted education, displacement-related inequalities, financial hardship, and limited access to social services.

The research team included one male and two female researchers, originally from Iran as well as UK-based, non-Iranian colleagues. Potential biases were mitigated by seeking broader supervisory input from HAMI staff experienced in conducting research studies with refugee families, ensuring a balanced and inclusive approach to recruitment and data analysis. Furthermore, close collaboration with HAMI as a local NGO and the authors' cultural diversity expanded our understanding of the context, practices, and norms when reporting the study findings.

Appendix S4. Ontological and epistemological orientation

Thematic analysis is considered a paradigmatically flexible analytical method and can be used across a range of ontological and epistemological positions (Braun & Clarke, 2006). Given the purpose of this study, we worked within an experiential qualitative orientation, which focuses on understanding participants’ lived experiences and their own accounts and interpretations, viewing language as a reflection of their internal understanding (Braun & Clarke, 2022). We adopted a critical realist ontological position, which conceptualises that reality exists and can be known (realism), but also holds that human action and language always shape what we experience and know (relativism). Our epistemological stance was phenomenological, which recognises first-person lived experience as a valid source for exploring, analysing, and contextualising how participants make sense of their own experiences (Braun & Clarke, 2022). Over the course of this study, our research team actively reflected on how their identities, expertise, values, and experiences in researching family mental health and developmental psychopathology informed the thematic analysis.

**Table S1***Details of HAMI researchers involved in interviewing and assessments*

| **Initials** | **Gender** | **Training** | **Education** | **Occupation** |
| --- | --- | --- | --- | --- |
| MY | Female | Post graduate training in research methods, qualitative and quantitative research including interviewing, and thematic analysis. Art therapy with children | BSc in Sociology, MSc in Psychology in progress | Research Assistant |
| EA | Female | Training in research methods during Bachelors' degree and education of refugee children | BSc in Social Sciences | Research Assistant |
| MM | Female | Mental health needs of refugee families, and supporting conflict-affected families | BSc, MSc in Psychology | Research Assistant |
| NS | Female | Training from BSc and MSc in qualitative research methods and data analysis  Psychotherapy with ethnically diverse families, including Afghan refugees  Training and delivery of focus groups, delivering interventions and data collection from interviews with children. | BSc, MSc, PhD in Clinical Psychology and Counselling | Senior Research |

**Table S2***COREQ criteria for Reporting Qualitative Research*

| **Item No** | **Guide Questions/Description** | **Responses** |  |  |
| --- | --- | --- | --- | --- |
| **Domain 1: Research team and reflexivity** | | |  |  |
| **Personal Characteristics** | | |  |  |
| 1. Interviewer/ facilitator | Which author/s conducted the interview or focus group? | The lead author (MR) frequently travelled to facilitate in the data collection process. All interviews were conducted by HAMI researchers. |  |  |
| 2. Credentials | What were the researcher’s credentials? E.g., PhD, MD | The lead researcher had completed a BSc and an MSc in Psychology prior to data collection and data analysis. |  |  |
| 3. Occupation | What was their occupation at the time of the study? | The researcher was a full-time PhD student at the time of data collection. |  |  |
| 4. Gender | Was the researcher male or female? | The researcher was male. |  |  |
| 5. Experience and training | What experience or training did the researcher have? | The researcher had completed two modules in qualitative data analysis during his PhD training courses, in addition to previous modules in qualitative and quantitative units in his BSc and MSc programmes. The researcher had extensive experience working with refugee families in Iran and other resource-poor settings. |  |  |
| **Relationship with participants** | | |  |  |
| 6. Relationship established | Was a relationship established prior to study commencement? | The lead researcher (MR) had already contacted the HAMI research team to facilitate the data collection and assessments. There was no relationship with participants prior to the study commencement. |  |  |
| 7. Participant knowledge of the interviewer | What did the participants know about the researcher? e.g. personal goals, reasons for doing the research? | Before the interviews were conducted, HAMI researcherers explained the study and their reasons for doing it to the participants. |  |  |
| 8. Interviewer characteristics | What characteristics were reported about the interviewer/facilitator? e.g. Bias, assumptions, reasons and interests in the research topic | All interviews were conducted by HAMI researchers. Bias was reduced by using independent qualitative researchers to code a subsection of the data. No assumptions were made by the lead and HAMI researchers prior to the study. More details about the interviewer and research team is provided in Table S2 and appendix S3. |  |  |
| **Domain 2: study design** | | |  |  |
| **Theoretical framework** | | |  |  |
| 9. Methodological orientation and Theory | What methodological orientation was stated to underpin the study? e.g. grounded theory, discourse analysis, ethnography, phenomenology, content analysis | The qualitative interviews were analysed using an inductive thematic analysis approach. |  |  |
| **Participant selection** | | |  |  |
| 10. Sampling | How were participants selected? e.g., purposive, convenience, consecutive, snowball | Participants were selected through purposive and snowballing sampling. |  |  |
| 11. Method of approach | How were participants approached? e.g., face-to-face, telephone, mail, email | In collaboration with HAMI NGO, we identified eligible participants via their local offices and networks with refugee families. More details can be found in the Methods (see pages 6-12). |  |  |
| 12. Sample size | How many participants were in the study? | Thirty refugee parents. |  |  |
| 13. Non-participation Setting | How many people refused to participate or dropped out? Reasons? | Of the 84 potentially eligible refugee parents initially approached by HAMI researchers, 51 participants declined or did not attend the scheduled interview slot. Additionally, a number of participants (n = 3) requested to withdraw their data within three weeks of their participation, due to personal reasons. see Figure S1 for the recruitment process. |  |  |
| 14. Setting of data collection | Where was the data collected? e.g., home, clinic, workplace | The interviews were carried out either at the HAMI local offices in Tehran, Semnan, and Mashhad. |  |  |
| 15. Presence of nonparticipants | Was anyone else present besides the participants and researchers? | No |  |  |
| 16. Description of sample | What are the important characteristics of the sample? e.g. demographic data, date | The most important characteristics of the sample was the literacy level of the participants, with 83% being illiterate. The interviews were conducted between April and November 2023. |  |  |
| **Data collection** | | |  |  |
| 17. Interview guide | Were questions, prompts, and guides provided by the authors? Was it pilot tested? | Interview questions and prompts were co-created by the authors team and HAMI team, and can be found in the supplementary file. The interview was pilot tested within the research team prior to data collection. |  |  |
| 18. Repeat interviews | Were repeat interviews carried out? If yes, how many? | No, only one interview per participant was carried out in the present study. |  |  |
| 19. Audio/visual recording | Did the research use audio or visual recording to collect the data? | Only audio recording. |  |  |
| 20. Field notes | Were field notes made during and/or after the interview or focus group? | During the first author’s fieldwork, field notes were taken and reflections were recorded after visiting the HAMI researchers and data collection sites. |  |  |
| 21. Duration | What was the duration of the interviews or focus group? | The length of the interviews ranged from 22 to 73 minutes depending on participants' ability to expand on their answers, with the average length of the interviews being 37 minutes. |  |  |
| 22. Data saturation | Was data saturation discussed? | Data saturation was reached after the 28^th^ interview. During a group discussion, it was agreed between authors and the lead researcher to conduct two-four additional interviews. |  |  |
| 23. Transcripts returned | Were transcripts returned to participants for comment and/or correction? | Transcripts were not returned to participants as no participant requested to see their transcript. All transcripts were transcribed by the lead researcher (MR) and HAMI qualified transcribers. |  |  |
| **Domain 3: analysis and findings** | | |  |  |
| **Data analysis** | | |  |  |
| 24. Number of data coders | How many data coders coded the data? | Two. The lead researcher (MR) and another independent qualitative researchers (FB). |  |  |
| 25. Description of the coding tree | Did the authors provide a description of the coding tree? | N/A |  |  |
| 26. Derivation of themes | Were themes identified in advance or derived from the data? | The themes were derived from the data as an inductive approach to thematic analysis was taken. |  |  |
| 27. Software | What software, if applicable, was used to manage the data? | N/A |  |  |
| 28. Participant checking | Did participants provide feedback on the findings? | Yes, preliminary results were presented to an advisory group of refugee parents by HAMI team. |  |  |
| **Reporting** | | |  |  |
| 29. Quotations presented | Were participant quotations presented to illustrate the themes/findings? Was each quotation identified? e.g., participant number | Quotations are provided throughout the results section to illustrate the identified themes and sub-themes. Each quotation is attributed to the participant’s gender, age, country of origin, and number of children. |  |  |
| 30. Data and findings consistent | Was there consistency between the data presented and the findings? | The themes were derived from the data, ensuring consistency between the data and the findings presented. Data extracts were selected to illustrate the themes discussed in the findings. |  |  |
| 31. Clarity of major themes | Were major themes clearly presented in the findings? | Yes, the four overarching themes are evident within the results and discussion section. |  |  |
| 32. Clarity of minor themes | Is there a description of diverse cases or a discussion of minor themes? | Each overarching theme has two to four candidate sub-themes which are briefly discussed. |  |  |

Developed from: Tong A, Sainsbury P, Craig J. Consolidated criteria for reporting qualitative research (COREQ): a 32-item checklist for interviews and focus groups. *International Journal for Quality in Health Care*. 2007. Volume 19, Number 6: pp. 349 – 357

**Table S3**Summary of trauma types experienced as measured by the PDS-5

| Trauma type | *Experienced (N=30)* | | *Focal (N = 30)* | |
| --- | --- | --- | --- | --- |
|  | *n* | *%* | *n* | *%* |
| Life threatening illness | 12 | 40 | 3 | 10 |
| Physical Assault | 16 | 53 | 3 | 10 |
| Sexual assault | 1 | 3 | 0 | 0 |
| Military combat or lived in a war zone | 27 | 90 | 21 | 70 |
| Child abuse | 6 | 20 | 1 | 3 |
| Serious accident | 7 | 23 | 1 | 3 |
| Natural disaster | 6 | 20 | 1 | 3 |

*Note*. Focal = the most distressing event discussed by the parent in the qualitative interview

**Table S4**
Summary of trauma types experienced reported by UCLA-RI parent version

| Possible child trauma exposures | *Experienced (n = 30)* | | *Focal (n = 30)* | |
| --- | --- | --- | --- | --- |
|  | *n* | *%* | *n* | *%* |
| Natural disaster | 7 | 23 | 1 | 3 |
| Serious accidental injury | 4 | 13 | 4 | 13 |
| War or forced displacement | 28 | 93 | 14 | 46 |
| Physical abuse | 12 | 40 | 0 | 0 |
| Witnessed domestic violence | 12 | 40 | 0 | 0 |
| Physical assault | 7 | 23 | 0 | 0 |
| Witnessed community violence | 16 | 53 | 0 | 0 |
| Witnessed dead body | 12 | 40 | 0 | 0 |
| Sexual abuse by a caregiver/parent figure | 0 | 0 | 0 | 0 |
| Heard about the violent death of a loved one | 9 | 30 | 10 | 33 |
| Painful medical treatment | 9 | 30 | 0 | 0 |
| Trafficking/sexual exploitation | 2 | 6 | 1 | 3 |
| Bereavement | 22 | 73 | 0 | 0 |

*Note.* Focal = the experience identified by the parent as the child’s most distressing in the qualitative interview

**Table S5***Summary of focal traumas and PTSD symptom scores for each participant*

| **PID** | **Index trauma** | **Description of the parental focal trauma** | **PDS-5 score** | **PDS severity** | **Description of the child's focal trauma** |
| --- | --- | --- | --- | --- | --- |
| 001 | Physical assault | Attack when Taliban soldiers were present | 44 | Severe | Two Taliban soldiers killed a 10 year-old child in front of her |
| 002 | War | Living during war and armed conflict | 31 | Moderate | War |
| 003 | War | Living during war and armed conflict | 48 | Severe | She witnessed an accident in which someone was severely injured |
| 004 | Life-threatening illness | Having a heart attack | 26 | Moderate | Her husband’s maternal cousin was martyred/killed by the Taliban, and she saw the body in a shroud |
| 005 | Life-threatening illness | Surgery in hospital | 53 | Severe | She witnessed a severe accident where dead people were lying on the ground |
| 006 | War | Did not described | 34 | Moderate | Living in a war-affected environment |
| 007 | Life-threatening illness | Has had a serious illness | 53 | Severe | She saw people killed by the Taliban lying on the ground |
| 008 | War | Her husband’s cousin was dismembered by the Taliban, and she witnessed it | 44 | Severe | He witnessed someone being hanged by the Taliban |
| 009 | Physical assault | She was attacked in their neighbourhood | 47 | Severe | His father was injured in a war with the Taliban, and he witnessed his father’s war injury |
| 010 | War | Witnessing an arrest and the Taliban being present in the home | 39 | Moderate | She was taken from the home by force by a Taliban soldier |
| 011 | War | Being in a war situation [exposure to wartime conditions] | 33 | Moderate | Sudden separation from her sister and brothers because of war |
| 012 | War | NA | 35 | Moderate | Fear of war and the Taliban attacking the home |
| 013 | War | Her husband was tortured due to political conflict/involvement | 32 | Moderate | Her brother was killed |
| 014 | War | Fear of war and frequent gunfire sounds | 61 | Very severe | Death of her sister and father |
| 015 | War | Living in wartime conditions | 48 | Severe | The martyrdom/death of one of her brothers |
| 016 | War | Bad memories of war and having been in it [being present during war] | 37 | Moderate | The martyrdom/death of his sister |
| 017 | War | Severe fear of Taliban war/attacks | 40 | Severe | Sudden death of his brother |
| 018 | War | Taliban attacked the home and forced them out of the house | 33 | Moderate | Taliban attacked the home and cut the child’s ear |
| 019 | War | Taliban attacked and fired at the home and demanded food | 37 | Moderate | Witnessing Taliban shot her brother |
| 020 | War | Her husband was shot during the war | 49 | Severe | Killing of her brother |
| 021 | War | Taliban attacked her husband and arrested him | 34 | Moderate | A very severe accident in childhood |
| 022 | War | No further explanation provided | 34 | Moderate | His father suffered burns in an accident |
| 023 | War | Witnessing an attack on the home to arrest her husband | 33 | Moderate | Death of her brother in an accident |
| 024 | War | Witnessing the arrest of her children during the war [children taken/detained] | 39 | Moderate | Martyrdom/death of her son; she witnessed the killing of her neighbour who was her close friend |
| 025 | Disaster | Experiencing a severe earthquake | 40 | Moderate | Sudden martyrdom/death of his husband |
| 026 | War | Severe fear of Taliban attacks and shootings in their place of living | 44 | Severe | Being in a war situation [exposure to wartime conditions] |
| 027 | War | Their home/place of living caught fire, and they fled from the Taliban | 34 | Moderate | Severe fear of war |
| 028 | War | Being in a war situation [exposure to wartime conditions] | 33 | Moderate | Sudden death of her brother |
| 029 | War | Was hit by shrapnel during the war | 34 | Moderate | Taliban attacked the child’s bedroom |
| 030 | Physical assault | Severe conflict and intense fighting/arguments [serious dispute; could be physical or verbal] | 42 | Moderate | He was severely injured in an earthquake |

*Note.* PID: Participant identification number; PDS: Posttraumatic Stress Diagnostic Scale.

**Table S6**
Themes and subthemes following thematic analysis

| **Theme** | **Subtheme** |
| --- | --- |
| 1. Parents’ experience in understanding and interpreting children’s distress | 1.1. Uncertainty about when and how to provide support |
|  | 1.2. Little understanding of mental health as a concept |
|  | 1.3. Support provided for children’s physical symptoms only at crisis point |
|  | 1.4. Spiritual resilience: a resource for meaning-making and recovery |
| 2. Parenting under displacement: depleted resources and inherited practices | 2.1. Parenting is learnt intergenerationally, not taught |
|  | 2.2. Chronic pain and health problems impair caregiving ability |
|  | 2.3. When post-resettlement stressors provoke harsh parenting |
| 3. Intra-family dynamics | 3.1. Communication gaps in understanding children’s support needs |
|  | 3.2. Children take on parenting-like responsibilities within the family |
| 4. Support needs, help-seeking, and care pathways | 4.1. Meeting children’s basic needs outranks all other support needs |
|  | 4.2. Protective role of informal support networks |
|  | 4.3 Accessibility, affordability, and helpfulness of psychosocial services |

**Figure S1**
*Flowchart of the participant recruitment process*


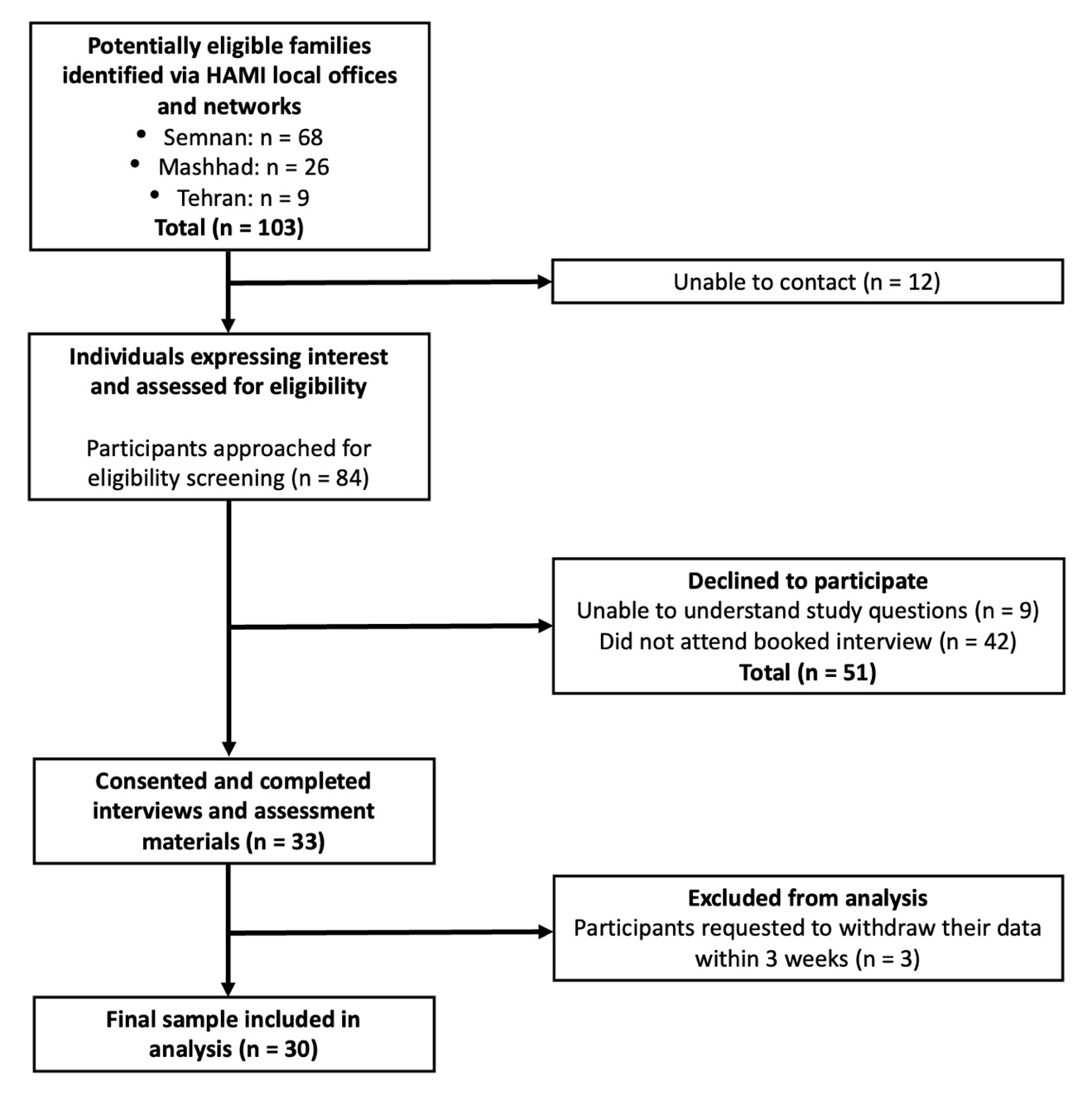

Supplement: Supplementary File_28226_R1.docx [file ZEPT_A_2693362_SM1000.docx]
